# Factors associated with accidents involving biological material among health professionals

**DOI:** 10.47626/1679-4435-2022-994

**Published:** 2024-02-16

**Authors:** Leila de Fátima Santos, Gleisy Kelly Neves Gonçalves, Soraya Rodrigues de Almeida Sanches, Wanessa Trindade Clemente

**Affiliations:** 1 Universidade Federal de Minas Gerais, Belo Horizonte, MG, Brazil; 2 Faculdade Ciências Médicas de Minas Gerais, Belo Horizonte, MG, Brazil

**Keywords:** health personnel, penetrants, professional exposure, risk factors, underreporting, pessoal de saúde, ferimentos penetrantes, exposição profissional, fatores de risco, subnotificação

## Abstract

**Introduction:**

Accidents with biological material and cuting/piercing instruments among health professionals have led to increased rates of hospital infection and subsequent patient contamination.

**Objectives:**

To compare factors associated with accidents involving biological material among health workers.

**Methods:**

This cross-sectional epidemiological study, conducted in 2019-2020, included 229 physicians and non-physicians.

**Results:**

The sample was 60.7% physicians and 39.3% non-physicians; 51.5% were women; 48.5% were aged ≥40 years; 55% lived with a partner; 57.6% had a specialist or graduate degree; and 51.5% had ≥ 1 child). he physician group had a higher education level, worked > 1 job, and had a high rate of accidents, in addition to lower rates of pre-employment examinations, specific accident training, and supervisor contact in case of accidents. There was also a positive association in the physician group between accidents, employment length, and operating room experience, while age was inversely correlated with accident risk.

**Conclusions:**

Different worker categories had specific risk profiles that involved education level, employment length, a low notification level, and risk underestimation. The results showed that education level and employment length do not guarantee accidents prevention. Both the physician and non-physician groups had significant accident rates and a similar behavior profile when events occurred, including low notification rates and underestimating the risk involved in the accident.

## Abstract (pt)

**Introdução:**

Acidentes envolvendo material biológico com instrumentos cortantes e perfurantes entre profissionais de saúde têm sido causa de aumento de infecção hospitalar e, portanto, de contaminação do paciente.

**Objetivos:**

Comparar fatores associados ao acidente com material biológico em trabalhadores da saúde.

**Métodos:**

Estudo epidemiológico transversal envolvendo 229 profissionais, médicos e não médicos (2019-2020).

**Resultados:**

A amostra total foi composta por 229 profissionais (48,1% médicos, 51,9% não médicos, 51,5% mulheres, 48,5% ≥ 40 anos, 55% viviam com companheiro, 57,6% tinham nível educacional de especialização/pós-graduação, 51,5% ≥ um filho). Os profissionais médicos apresentaram maior escolaridade, mais de um emprego e maior proporção de ocorrência de acidentes, bem como menor proporção de exames admissionais, treinamentos específicos e contato com supervisor em casos de acidentes. Ainda, os profissionais médicos apresentaram associação positiva dos acidentes com o tempo de trabalho e o tempo de experiência no bloco cirúrgico, enquanto a idade apresentou relação inversa com as chances de acidentes.

**Conclusões:**

Diferentes categorias de trabalho apresentaram perfil específico de riscos envolvendo estudo e tempo de serviço, baixa notificação e risco subestimado. Os resultados deste estudo mostraram que o nível de estudo e o tempo de serviço não foram capazes de garantir a prevenção de acidentes envolvendo material biológico. Além disso, profissionais médicos e não médicos apresentaram não apenas uma incidência significativa de acidentes, mas também um perfil de comportamento semelhante diante do evento, com baixa notificação e risco subestimado do acidente.

## INTRODUCTION

Accidents with biological material and cuting/piercing instruments among health professionals have led to increased rates of hospital infection and subsequent patient contamination. Exposure is defined as direct or indirect contact with human blood or biological fluids that involves a potential degree of contamination. These accidents generally occur while nurses and physicians handle cuting and piercing instruments, given the high frequency of use during invasive procedures.^1,2^

Different types of care entail specific risks based on the involved tasks. Professionals who work in operating rooms or surgical centers are exposed to physical, chemical, and, chiefly, biological risks.^1^ Occupational accidents can be serious, with outcomes including infection and death.^3^ Sharps, specifically needles, are considered extremely dangerous since they can transmit pathogens. Hepatitis B, hepatitis C, and HIV are the main contaminants associated with these instruments.^1,4^ These risks are further increased in non-immunized workers, who must be tested and included in a post-exposure prophylaxis program that includes complementary vaccination for viral hepatitis and tetanus.

It is important to determine which factors contribute to accidents with biological material.^5^ To promote initiatives and training to reduce these events, professional and institutional characteristics must be investigated.^6^

The present study aimed to determine and compare factors associated with accidents involving biological material among physicians and non-physicians at a large public hospital in Belo Horizonte, Minas Gerais, Brazil.

## METHODS

### SETTING AND DESIGN

This quantitative epidemiological, cross-sectional study investigated biological risk among health professionals during surgical procedures. The study was conducted at a general university hospital that exclusively treats users of the Brazilian Unified Health System (Sistema Único de Saúde). The hospital plays a fundamental role in handling urgent/emergency cases in the municipal network, treating clinical and trauma emergencies and performing a wide variety of surgical procedures, all of which involve contamination potential. The hospital has an infection control department that complies with state and municipal health service regulations and U.S. Centers for Disease Control and Prevention criteria.

### STUDY POPULATION AND PROCEDURES

The convenience sample consisted of physicians of different specialties, oral and maxillofacial surgeons, nurses, nursing assistants, and nursing technicians. Employees present on data collection days were invited to participate in the study. Since all of them accepted, there was no sample loss. The participants were divided into 2 groups, physicians and non-physicians, to facilitate comparative analysis of the findings. Oral and maxillofacial surgeons and nursing staff (nurses and nurse technicians) were considered non-physicians.

Data were collected between 2019 and 2020 by previously trained researchers using the instrument “Comply with post-exposure management among health care workers”, adapted from Jansen (2014). The instrument consists of 47 questions, including demographic and occupational exposure variables, in addition to follow-up and post-exposure prophylaxis. This study was approved by the institutional research ethics commitee (number 57295816.6.0000.5149) in compliance with national resolution 466/2012 regarding research involving human beings.

### STATISTICAL ANALYSES

Descriptive statistics were presented as frequencies and proportions; univariate analysis included the chisquare test of independence or Fisher’s exact for each variable between physician and non-physician groups. Te Shapiro-Wilk normality test was also applied. “I don’t know” responses were considered missing data. Te Wilcoxon Mann-Whitney test was used to compare groups of professionals. Binary logistic regression was used to assess factors associated with the occurrence of accidents involving biological material. Two different models were built: one for physicians and another for non-physicians; the data were presented as odds ratio and p-value.

Variables with p < 0.20 in the univariate analysis were included in a full model, and a backward stepwise approach was used to arrive at the final model, which retained variables with p < 0.05. Te results are presented as odds ratio and 95%CI. The analyses were performed in R version 4.0.2, with p < 0.05 considered significant.

## RESULTS

### DESCRIPTIVE ANALYSIS OF THE SAMPLE

The total sample consisted of 229 professionals, of whom 60.7% were physicians and 39.3% were non-physicians. A total of 51.5% were women, 48.5% were ≥ 40 years of age, 55% lived with a partner, 57.6% had graduate degree, and 51.5% had ≥ 1 child.

### WORK-RELATED CHARACTERISTICS BY PROFESSION

In the physician group, most worked ≤24 hours per week (p < 0.001); there were significant differences between those who worked day and night shifts (p < 0.001), in the emergency surgical center (p < 0.001), in the obstetric surgical center (p = 0.041), in other health institutions (p < 0.001), and in those who reported a previous accident with biological material (p = 0.007) (Table 1). More than half of the non-physician group reported: a weekly workload between 24 and 40 hours, working the day shift, working in the urgent surgical center, and not working at other health institutions.

**Table 1:** Work characteristics according to professional group

Characteristics	Physicians (n = 139)	Non-physicians (n = 90)	p-value	Total (n = 229)
	n (%)	n (%)		n (%)
Weekly workload at the hospital* (hours) (n = 227)			<0.001†	
≤24	81 (59.6)	4 (4.4)		85 (37.4)
25-40	5 (3.7)	59 (64.8)		64 (28.2)
>40	50 (36.8)	28 (30.8)		78 (34.4)
Work shift at the hospital* (n = 227)			<0.001†	
Day	51 (37.5)	57 (62.6)		108 (47.6)
Night	14 (10.3)	22 (24.2)		36 (15.9)
Other	71 (52.2)	12 (13.2)		83 (36.6)
Hospital sector ‡				
Elective surgery	69 (49.6)	41 (44.6)	0.534†	110 (47.6)
Urgent surgery	116 (83.5)	54 (58.7)	<0.001†	170 (73.6)
Obstetric surgery	23 (16.5)	6 (6.5)	0.041†	29 (12.6)
Jobs at other health institutions (n = 229)	114 (82)	34 (37.7)	<0.001†	148 (64.6)
1	26 (22.8)	24 (70.6)		50 (33.8)
**>**2	88 (77.2)	10 (29.4)		98 (66.2)

Values in bold showed a statistically significant difference.

*Variables with missing values.

^‡^Variable allowed multiple responses.

^†^Chi-square test.

The physician group had a lower rate of pre-employment examinations (p < 0.001), specific training about accident prevention and post-accident procedures (p = 0.004), and supervisor contact in case of exposure to biological materials (p = 0.005) (Table 2). Pre-employment examinations were quite common (90%) in the non-physician group, while 61.9% received specific training on accident prevention and post-accident procedures; 74.4% consulted a supervisor after accidents.

**Table 2:** Accident prevention habits according to professional group

Characteristics	Physicians (n = 139)	Non-physicians (n = 90)	p-value	Total (n = 229)
	n (%)	n (%)		n (%)
Frequency of PEP*			0.488†	
Always/almost always	130 (94.9)	87 (97.8)		217 (96.0)
Rarely/never	7 (5.1)	2 (2.2)		9 (4.0)
Vaccinated against hepatitis B*	135 (97.8)	91 (100.0)	0.278†	226 (98.7)
Anti-hepatitis B test after vaccination*	126 (92)	76 (90.5)	0.891‡	202 (91.4)
Vaccination card requested upon admission*	108 (87.1)	83 (94.3)	0.104‡	191 (90.1)
Underwent pre-employment examinations*	108 (78.3)	87 (95.6)	<0.001†	195 (85.2)
Specific training on accident prevention and post-accident procedures*	47 (40.2)	52 (61.9)	0.004‡	99 (49.3)
Frequency of occupational health evaluation* (n = 191)			0.060‡	
Semi-annually	9 (7.5)	7 (9.9)		16 (8.4)
Annually/biannually	51 (42.5)	41 (57.7)		92 (48.2)
Never	60 (50)	23 (32.4)		83 (43.5)
First contact after exposure to biological materials*			0.005‡	
Supervisor	71 (52.2)	67 (74.4)		138 (61.1)
Infection control/CCIH/SCIH	21 (15.4)	5 (5.6)		26 (11.5)
Occupational safety and health	20 (14.7)	6 (6.7)		26 (11.5)
Other	24 (17.6)	12 (13.3)		36 (15.9)
Previous accident with biological material *	64 (48.1)	27 (29.3)	0.007‡	91 (40.4)

Values in bold showed a statistically significant difference.

*Variable with missing values.

^†^Fisher’s exact test.

^‡^Chi-square test.

CCIH/SCIH = Comissão de Controle de Infecção Hospitalar/Serviço de Controle de Infecção Hospitalar; PEP = post-exposure prophylaxis.

### FACTORS ASSOCIATED WITH ACCIDENTS INVOLVING BIOLOGICAL MATERIAL AMONG PHYSICIANS AND NON-PHYSICIANS

In the univariate analysis, none of the variables were significantly associated with the occurrence of accidents. In the multivariate model, 6 to 15 years of employment at the hospital (p = 0.014) and working in the elective surgery center (p = 0.042) were associated with a higher risk of accidents, while age between 30 and 39 years (p = 0.022) was associated with a lower accident risk in the physician group.

In univariate analysis of the non-physician group, there was a higher risk of accidents among those without a partner (p = 0.017), those with a second job (p = 0.015), and those who had been employed between 6 and 15 years (p = 0.043) or ≥16 years (p = 0.014) at the hospital. Having ≥1 children was associated with a lower accident risk (p = 0.005).

Similar results were found in the multivariate model: having no partner (p = 0.010) and employment length between 6 and 15 years (p = 0.007) or ≥16 years (p = 0.013) at the hospital were associated with a higher accident risk, while having ≥1 children (p = 0.030) and reporting awareness of the hospital’s exposure notification rules (p = 0.023) were associated with a lower accident risk (Table 3).

**Table 3:** Factors associated with accidents involving biological materials among hospital employees

Characteristics	Physicians (n = 139)				Non-physicians (n = 90)			
	Univariate models		Multivariate model		Univariate models		Multivariate model	
	OR (95%CI)	p-value	OR (95%CI)	p-value	OR (95%CI)	p-value	OR (95%CI)	p-value
Sex, male (ref. female)	0.56 (0.27-1.14)	0.113	-	-	1.14 (0.36-3.32)	0.811	-	-
colspan="9">Age group (ref. < 30 years)
30 to 39	0.40 (0.15-1.08)	0.074	0.28 (0.09-0.82)	**0.022**	0.78 (0.13-69.36)	0.797	-	-
≥ 40	0.72 (0.26-1.93)	0.518	0.20 (0.04-1.00)	0.053	0.84 (0.15-6.15)	0.851	-	-
Marital status (ref. no partner)	0.56 (0.27-1.13)	0.1 07	-	-	3.33 (1.28-9.50)	**0.017**	13.04 (2.21-128.08)	**0.010**
colspan="9">Education (ref. high school/technical course)
Undergraduate degree					1.69 (0.57-5.02)	0.337		
Graduate degree	1.53 (0.67-3.57)	0.314	-	-	1.60 (0.52-4.86)	0.405	-	-
colspan="9">Number of children (ref. O)
≥1	1.56 (0.79-3.12)	0.205	-	-	0.26 (0.10-0.66)	**0.005**	0.17 (0.03-0.78)	**0.030**
colspan="9">Employment type (ref. hired man)
Civil servant	0.93 (0.36-2.36)	0.876	-	-	1.84 (0.55-7.32)	0.346	-	-
Other	0.71 (0.28-1.81)	0.474	-	-	6.11 (1.50-29.21)	**0.015**	-	-
colspan="9">Time employed in health care (ref. ≤10 years)
11 to 20	1.16 (0.47-2.88)	0.747	-	-	1.16 (0.47-2.88)	0.747	-	-
≥2	0.88 (0.36-2.14)	0.783	-	-	0.88 (0.36-2.14)	0.783	-	-
Time employed at the hospital (ref. ≤5 years)								
6 to 15	2.04 (0.91-4.63)	0.084	4.54 (1.41-16.16)	**0.014**	3.08 (1.07-9.76)	**0.043**	16.70 (2.72-175.55)	0.007
>16	1.71 (0.70-4.21)	0.237	4.67 (0.97-24.70)	0.060	1.71 (1.43-22.63)	**0.014**	25.79 (2.43-462.89)	**0.013**
Weekly workload at the hospital (ref. ≤ 24h)								
25 to 40	1.58 (0.25-12.49)	0.627	-	-	012 (0.01-1.05)	0.080	-	-
>40	0.85 (0.41-1.76)	0.662	-	-	0.11 (0.01-1.02)	0.075	-	-
Shift at the hospital (ref. day)								
Night	0.64 (0.19-2.11)	0.463	-	-	2.34 (0.81-6.75)	0.112	-	-
Other	0.69 (0.33-1.44)	0.324	-	-	1.69 (0.40-6.34)	0.445	-	-
Hospital work sector								
Elective surgery center	1.89 (0.95-3.79)	0.070	2.16 (1.04-4.60)	**0.042**				
Urgent surgery center	0.59 (0.23-1.48)	0.263	-	-				
Obstetric surgery center	2.33 (0.93-6.22)	0.076	-	-				
Second job at another health institution?	1.44 (0.58-3.76)	0.438	-	-				
Frequency of PEP (ref. always/almost always)								
Rarely/never	2.84 (0.59-20.39)	0.222	-	-	2.78 (0.11-72.32)	0.476	-	-
Vaccinated against hepatitis B	0.46 (0.02-4.94)	0.533	-	-	-	-	-	-
Vaccination card required upon employment	0.69 (0.23-2.00)	0.496	-	-	0.23 (0.03-1.45)	0.116	0.09 (0.01-1.10)	0.056
Pre-employment examinations required	0.77 (0.33-1.78)	0.545	-	-	0.12 (0.01-0.99)	0.072	-	-
Participated in specific training about accident prevention and post-accident procedures	0.67 (0.31-1.43)	0.304	-	-	0.57 (0.21-1.53)	0.262	-	-
Frequency of periodic occupational health assessment (ref. biannual)								
Annual/biennial	1.42 (0.33-7.40)	0.641	-	-	3.11 (0.47-61.82)	0.315	-	-
Never	2.00 (0.48-10.20)	0.358	-	-	1.67 (0.21-35.30)	0.669	-	-
Awareness of the hospital’s notification procedures after biological material exposure	-	-	-	-	0.17 (0.01-1.84)	0.154	0.02 (0.0004-0.50)	**0.023**
First contact after biological material exposure (ref. supervisor)								
Infection control	1.79 (0.64-5.15)	0.268	-	-	-	-	-	-
Occupational safety and health	0.95 (0.33-2.63)	0.915	-	-	-	-	-	-
Other	1.42 (0.55-3.70)	0.469	-	-	-	-	-	-

Values in bold showed a statistically significant diference. Multivariate model: Hosmer-Lemeshow test p-value = 0.529 for physician group and 0.406 for non-physician group. The variable “Vaccination card required upon employment” was retained to ensure the convergence of the model. OR = odds ratio; PEP = post-exposure prophylaxis.

## DISCUSSION

We found that physicians had a higher accident risk than other professionals with a graduate degree or workers with significant employment length at the hospital. Age was inversely related with accident risk. Working in the elective surgery center was also associated with accidents. Tere was a higher proportion of men and a higher education level among physicians. Most physicians had ≥1 job and had suffered previous accidents. Pre-employment examinations were less often required of physicians, and they had less accident training and contact with supervisors after an accident.

A study of 901 health professionals at a Chinese hospital revealed that 27.5% suffered an acute injury in 2017. Seniority, position, title, education, department, and training programs were associated with the occurrence of sharps injuries. The most elaborate statistical approach indicated that seniority and training programs were most closely associated with the occurrence of acute injuries.^7^ Similar to our results, the authors found that only 33.9% of workers reported their injuries to the appropriate authorities. Cui et al.^7^ found that the main reasons for not reporting sharps injuries were perceiving the injury to be insignificant and worker immunization status.

Based on these findings, it would seem that worker habits and behavior determine professional conduct when accidents occur, making post-injury control and follow-up more difficult. Another study found that occupational accidents occurred more frequently among nurses than physicians and that age > 40 years was associated with accidents.^4^

A cross-sectional study investigated the profile of occupational accidents among 47,629 participants in the Brazilian National Health Survey. Accidents were associated with intense noise, biological materials, work experience ≥ 40 years, and intense physical exertion.^8^ In our study, employment length and exposure to biological materials were closely associated with accidents, which reinforces the high level of risk among health care workers and suggests that precaution should be proportional to the risk level.

In the non-physician group, the occurrence of accidents was higher among those without a partner, those who had a second job, and those with greater employment length at the hospital. Having children and being aware of the hospital’s accident notification rules were also negatively associated with accident risk. Other studies of Brazilian health workers have also found an association between employment length, age, and accidents involving biological material. One study^9^ interviewed 226 nursing professionals from a large hospital in the state of São Paulo, finding that 17.3% had reported occupational exposure to biological material and that most accidents involved percutaneous contamination.

Because the worker profile and errors involved in accidents with biological material seem similar across health care institutions, accident prevention should be mandatory in professional training and built into health service routines.

Among non-physicians in the preset study, exposure to biological material occurred mainly percutaneously, eg, through puncture wounds by needles or drill bits during surgical procedures. Although the majority of workers reported to the hospital’s occupational health and safety department afer an accident, it is important to note that even superficial wounds involve contamination potential, and workers in our sample reported not notifying supervisors about accidents they considered insignificant. Similar results have been found among health workers who suffered accidents involving biological material in the state of Goiás, Brazil.^10^ The injury site was generally the hand, with the most common protective equipment used at the time of the accident being masks and closed shoes. Of note, few professionals who followed up with the medical team afer an accident received psychological counseling, which is important even when no specific symptoms occur. Another study found a low accident notification rate and that accidents were associated with rushing, carelessness, needle recapping, and performing procedures without gloves; men and members of the nursing team had the highest accident rates.^11^

Among physicians in the present study, most accidents happened during surgical procedures while they wore a surgical mask, apron, protective clothing, and double-layered gloves. Physicians had the lowest rate of notiflcations to and treatment by the occupational safety and health department, as well as follow-up by the occupational medicine team after accidents. Because these accidents can be fatal, the World Health Organization has developed a safety checklist for operating rooms. The purpose of the Surgical Safety Checklist, a 19-item tool created in association with the Harvard School of Public Health, is to reduce the occurrence of such events worldwide.^12^ Among physicians, there was lower awareness that accidents must be immediately reported to hospital authorities, which suggests that misinformation is a critical point among these professionals.

In some countries, reporting occupational accidents is a joint action by employee and employer.^13^ In Belo Horizonte and, specifically, the hospital involved in this study, accident notification is initiated by the affected worker, which can lower the number of reported accidents.

Given that this study was conducted at a single hospital, caution is needed when generalizing its findings. However, because it is a large hospital and many participants also held jobs at other hospitals, the results should represent the regional profile. A more robust study must be conducted for a more accurate understanding of factors related to accidents with biological material.

## CONCLUSIONS

The results of this study showed that education level and employment length did not guarantee accident prevention. Physicians and non-physicians not only had a significant incidence of accidents but behaved similarly when they occurred, including suboptimal notification habits and risk underestimation. The medical service was judged to be generally satisfactory. Nevertheless, confusion about notification procedures and care flow was observed. Such results indicate a risk scenario requiring decisive action from health workers to ensure good safety practices.
